# A Manual of the Nervous Diseases of Man

**Published:** 1844-04

**Authors:** 


### ART. IX.

*Lehrbuch des Nervenkrankheiten des Menschen.* Von M. H. ROMBERG, Doctor der Medicin, &c. Erster Band.—*Berlin,* 1840-43. 8vo, pp. 610.

*A Manual of the Nervous Diseases of Man.* By M. H. ROMBERG, Doctor of Medicine, Professor and Director of the Royal Polyclinical Institute of the University of Berlin, &c. Vol. I. Part I, 1840; Part II, 1843.

PROFESSOR ROMBERG is of opinion that the diseases of the nervous system should now be considered scientifically. He has been led to this conclusion from a consideration of the numerous researches instituted of late years by physiologists, and of the important results which have followed; and he has been induced to act on that opinion from the conviction that no work has yet been published in which the neuroses were so discussed. We need scarcely say that important as modern neurological discoveries confessedly are, the physiology of the nervous system is as yet undeveloped, and can only be but very imperfectly applied to pathology. There is more to be filled up than the gaps acknowledged by our author. Whole fields of research remain altogether unexplored, and we find that the further we advance the more we have to discover. This is not written with intent to disparage Professor Romberg's labours; on the contrary, we think that he has manfully grappled with a difficult subject, and we gratefully accept his work, to use his own phrase, as the first step in the right direction.

His classification of nervous diseases is founded on the four great functional divisions of the nervous system, namely, the sensitive, motor, mental, and nutrient. Upon these, four classes of diseases are formed, namely: 1. The Sensitive neuroses. 2. The Motor neuroses. 3. The Phreno-neuroses. 4. The Tropho-neuroses.

The first part of this, the first volume, is devoted to the Sensitive neuroses; the second to the Motor neuroses. The following are Professor Romberg's subdivisions of his first class—the Sensitive neuroses. The class divides naturally, according as the normal sensibility is increased or diminished, into Hyperesthesiæ and Anesthesiæ.

Order I.—Hyperesthesiæ of the Nerves.

A. *The Cerebro-spinal Nerves.*

Genus 1. *Cutaneous hyperesthesiæ.*
*a.* Neuralgia. *b.* Pruritus. *c.* Ardor. *d.* Algor.

Genus 2. *Muscular hyperesthesiæ.*
*a.* Neuralgia muscularis. *b.* Vertigo.

Genus 3. *Pueumo-gastric hyperesthesiæ.*
*a.* Neuralgia—Globus, Pyrosis, Gastrodynia neuralgica. *b.* Bulimia. *c.* Polydipsia.

Genus 4. *Sensual hyperesthesiæ.*
*a.* Hyperesthesia optica. *b.* H. acoustica. *c.* H. olfactoria. *d.* H. Gustatoria.

B. *Hyperesthesiæ of the Sympathetic Nerves.*

Genus 1. Hyp. plexus cardiaci.
,, 2. ,, solaris.
,, 3. ,, mesenterici.
,, 4. ,, hypogastrici.
,, 5. ,, spermatici.

Order II. Hyperesthesiæ of the Central Organs.

A. *Of the Spinal Cord.*
Neuralgia spinalis.

B. *Of the Brain.*
*a.* Neuralgia cerebralis. *b.* Hyperesthesia psychica.

The imperfections of this arrangement are obvious. In forming the second order, Professor Romberg abandons, with little ceremony, his own scientific principles, and adopts the old empirical method of arrangement. Neuralgia cerebralis is no neuralgia at all—in fact, no definite disease—but simply cephalæa, or violent headach, a *symptom* common to many and very different diseases, as we shall find as we proceed. Nor can we understand how vertigo is to be considered as hyperesthesia of the muscular nerves.

Order I. Hyperesthesiæ of the Nerves. The pathology of the sensitive neuroses must be founded on the physiological laws of sensation. There are three principal laws of this kind. The first is the law of isolated continuity of a nervous fibril and of isolated conduction of sensation from its peripheral to its central termination. This applies, without exception, to all the peripheral nerves. The second law is the law of irradiation of sensation in the central organ; and the third is the law of excentric phenomena, by which the sensations consequent upon change in a sensitive nerve in any part of its course are referred by the individual to its peripheral extremity. This law divides the hyperesthesiæ into the two divisions of peripheral and central, according as they are allocated in any part of the fibril in its course from the periphery to the centre, or in the central axis itself. This law is of very considerable importance in the study of the pathological anatomy of the sensitive neuroses, and in explaining the symptoms of diseases of the nervous system generally. In dissecting a case of crural neuralgia, for example, it would not be sufficient to confine the attention to the seat of pain. The whole course of the nerve must be traced up to its insertion in the spinal cord, and (if it were possible) through the cord, ganglia of the medulla oblongata, and the lower cerebral ganglia, to its termination in the hemispherical ganglia, supposing these to be the sensorium. By the second law of *radiation of sensation*, we understand how many sensations and motions originate. An impression in the peripheral end of a sensitive nerve, when communicated to the central axis, may there radiate: *a*, upon motor nerves, and give rise to *reflex* muscular notions; or *b*, upon sensitive nerves, and give rise to reflex or sympathetic sensations; *c*, upon a trophical nerve, and originate vascular congestion and secretion.* For example, a strong glare falling upon the eye will excite the reflex sensation of tickling in the nose, the reflex muscular action of sneezing, and reflex secretion from the lacrymal gland; all as strongly and powerfully, in many instances, as direct irritation of the Schneiderian membrane. In neuralgia of the ciliary nerve, the vessels of the conjunctiva are injected, the lacrymal gland excretes profusely, and the orbicularis palpebræ contracts spasmodically. It is thus that irritation of a single fibril extended to the central ganglia induces general convulsions. Other principles flow naturally out of these. The *act of attention* to impressions made on sensitive nerves renders the central termination of the impressed nerve more affectible, and removes it from its normal state in proportion as the attention is concentrated. And as this central change may radiate, and so act upon the trophical nerves of the part, as well as upon the motor and sensitive nerves, structural changes, and morbid sensations and movements may be induced, as occurs, for example, in hypochondriasis. The French metaphysician Bonnet was probably the first to develop this idea of the nature of attention. The phenomena of mesmerism, so called, are examples of the same kind. We have noticed this question in a previous Number. (vol. XIII, p. 13.)

It is manifest that the causes of hyperesthesiæ may be of the most varied character. Some of these are alluded to by Professor Romberg. The reflex action of a peripheral morbid structure on the central axis may, by the law of radiation, induce neuralgiæ; thus, sciatic neuralgia may result from disease of the rectum; pneumogastric hyperesthesia (as vomiting), from intestinal irritation. Poisons, or retained secretions acting as poisons, and acting upon specific portions of the central ganglia, are amongst the more frequent causes of neuralgia. Lead, for example, acting on the spinal cord, induces mesenteric neuralgiæ, acting on the brain induces epilepsy. Morphia, or retained bile, excites *pruritus;* secale cornutum and veratria bring on *formicatio*. The arthritic irritation dependent upon the presence of salts of urea in the blood, and usually concentrated upon the serous or sero-synovial membranes of joints and bursæ, may be diverted to the serous membranes of the central ganglia and of the larger nervous trunks, and so every form of neuralgia, indeed, every form of neurosis, may be developed, and with such activity as speedily to extinguish life; as, for example, when the serous coverings of the ganglia in connexion with the pneumogastric nerve or of the nerve itself are thus affected. Arthritis is mentioned by our author as a frequent cause of neuralgia, but he does not seem to have been at all impressed with the theory of its *modus operandi*, or he would undoubtedly have mentioned so important a matter, slightly at least. Mechanical

* The trophical nerves are distributed to the vascular and glandular systems; they correspond to the organic nerves of Müller.

irritants of every kind demand also a prominent position in the etiology of nervous diseases, and especially of the hyperesthesiæ. The pressure of an aneurism, or of the thickened parietes of a bony canal through which a nerve passes, or muscular action, or external irritants, may be mentioned as belonging to this class.

CUTANEOUS HYPERESTHESIÆ. This genus, containing true neuralgia, presents the best examples of the general laws just laid down, as it most frequently is the result of mechanical injury. Various illustrations are given, principally from English writers, as Abernethy, Wardrop, Denmark, and Swan. Subcutaneous tubercles and neuromatous tumours on the nerve, neuritis, hypertrophy, and ulceration from the proximity of cancerous sores, aneurisms, and tumours, are also noticed in detail. The treatment recommended in each case is precisely that generally adopted in England.

*Neuralgia nervi quinti.* This, the true tic douloureux, is considered at great length. Professor Romberg acknowledges that the facial nerve is sometimes the seat of pain, but combats the opinion advanced by Gädechens and Arnold, that it has a double function, and that the *portio intermedia* of Wrisberg (*portio minor*) constitutes the sensitive portion. This question we have lately discussed (see Br. and For. Med. Rev., vol. XIII, p. 148). The pathological anatomy of tic douloureux is well illustrated by the history of a case of twenty-six years' duration, commencing at the age of thirty-nine. Froriep conducted the post-mortem examination; we have not space, however, for the interesting result obtained by him. The physiological considerations arising out of it are not less worthy of notice than the anatomical, and are of greater importance, because they contribute to a more accurate diagnosis of this as well as other neuroses. The whole history illustrates the law of excentric phenomena previously stated. The cause of the neuralgia was within the cranium; yet there was no pain in the head, but only at the periphery of the nerve, namely, in the face. It also illustrates the neuro-physiological law of isolated conduction, applicable alike to sensitive and motor nerves. The *pars motoria*, although so near the morbid change in the sensory part, was altogether unaffected, and so also were the other cerebral and spinal nerves. Besides the neuralgia, the patient suffered, during the latter years of his life, from a sensation of vertigo and an inclination to fall over to the left side. This was accompanied with a feeling of weakness in the lower extremities. The fact is curious taken in connexion with the morbid condition of the affected nerve as it passed through the left *crus cerebelli.* It may be considered as a pathological experiment in corroboration of those of Majendie on living animals, in which section of a *crus cerebelli* was followed by a rotatory motion towards the same side. It is also a fact worthy phrenological notice, that although the brain was atrophied and the convolutions flattened, the mental powers were untouched to the last. The patient died of ischuria, and it was curious, too, that ten days before his death, the neuralgic affection disappeared never to return. Of course all treatment was vain; but the greatest ease was obtained by local depletion, occasional purgatives, and a strictly vegetable diet.

We have not space to follow our author through the etiology, diagnosis, prognosis, and treatment of the disease. And it is the less necessary we should do so, as there is little stated which is not to be found in our own authors. The diagnosis of tic douloureux from painful anesthesia is not always made and is not always necessary. In the latter, irritation of the skin easily induces a paroxysm, in the former it does not. There is one method of treatment, not mentioned by our author, to which we would allude, because we believe it is not generally known, and that is, to paralyse the nerve by keeping the patient continually narcotised for some days. In a long chronic case of true tic douloureux, iu which the agony had become most intense, utterly depriving the patient of rest, we were by this plan enabled to give so much *permanent* relief, that the patient was enabled to sleep for two or three hours continuously. We administered half a grain of acetate of morphia, and three grains of camphor every four hours for three or four days; at the end of which period the paroxysms became more endurable, and have continued so.

*Ciliary neuralgia.* This is the name given by our author to the affection popularly known as "weakness of sight." A douche twice daily to the eyes, continued for half an hour each time, with water impregnated with carbonic acid gas, is strongly recommended by Jüngken. Professor Romberg observes that he has applied a jet of the pure carbonic acid gas to the eyes in very obstinate cases with the best effect.

*Sciatic neuralgia.* As this is occasionally confounded with hip disease, we quote the following excellent diagnosis of the two affections :

"The pain is not confined to one part, but extends over the whole thigh, and in particular, the skin is more sensitive to pressure than the deep-seated parts. The patient complains more when the skin is nipped up and pinched, than when the head of the thigh-bone is pressed against the acetabulum. The pain is increased if the patient's attention be directed to the limb during the examination, but scarcely felt if he be engaged in agreeable conversation. There is no wasting of the glutæi, or flattening of the nates. The whole appearance indeed of the limb is unlike that accompanying disorganization of the joint. Sometimes the thigh and nates swell, and in some few cases there is a circumscribed tumour, but not such as to justify the suspicion of an abscess. There is no fluctuation, and the whole appearance of the tumour cannot be better compared to anything than an uncommonly large sting of a nettle. Although no deformity occurs from flattening of the nates, this does happen occasionally from a twisting of the pelvis backwards, so that at the same time it is higher on the affected side than the other, and forms an acute rather than a right angle with the vertebral column. The limb appears shortened in consequence, and in standing the heel does not touch the ground. There is sometimes a remarkable alternation of heat and cold, not merely in the joint but in the whole limb. In the morning it is cold, and of a pale livid colour; in the afternoon it is warm; in the evening hot, shining and congested. Spasmodic contractions of the affected limb are not unfrequent, and convulsive movements, not unlike those of chorea, are excited on pinching or lightly touching the integument. The limb is often jerked up to a considerable height by these spasms. In these cases, there is always a feeling of weakness, and this at last continues after the pain has remitted. Such a state will continue for weeks, months, and even years without any further bad consequences. The patients are almost always young girls, or females who menstruate irregularly, or are of the hysterical diathesis. Hysterical attacks indeed often precede or follow the disease with alternate remissions and returns. Sometimes some severe disease has preceded, and left the patient exhausted in body and depressed in mind." (pp. 66-7.)

*Treatment of sciatic neuralgia.* When the affection occurs in stout men as an acute inflammatory disorder, local and general bleeding is necessary. In other instances, the causes must indicate the mode of treatment. To ascertain these, the pelvic viscera should be carefully scrutinised to discover if there be any condition favorable to a morbid reflex or radiate action on the affected nerve as it traverses the spinal cord. Masses of indurated fæces in the bowels, wedging of the child's head on the pelvis, congestion or irritation of the uterus after parturition, are among the local causes. The general causes are too many to enumerate. The practitioner is, however, often unable to determine the cause, and then empirical treatment becomes necessary. The medicinal use of spirits of turpentine is strongly recommended by Professor Romberg. For his mode of administering it, see vol. XIII of this Journal, p. 157. He has also found repeated blisters to the outer side of the knee, and kept open for a few days, very useful in inveterate cases. As a palliative, from one quarter to half a grain of acetate of morphia may be sprinkled on the blistered surface.

*Crural neuralgia.* This neuralgia, like the sciatic, is very liable to be mistaken for disease of the knee-joint. We have suffered from it ourselves, and more than one surgeon has declared us to be labouring under incipient disease of the knee-joint. We have also met with two such cases in practice. A prognosis of this kind is exceedingly distressing, particularly to the medical patient, who can foresee clearly the course of a "white swelling" through all its stages of suffering to amputation and death. The general remarks on the sciatic neuralgia are applicable here; in addition it may be observed that the pain is quite circumscribed, and the afflicted part exquisitely sensible. In our own case, the sensitive point is a little spot on the outer edge of the patella. There is sometimes a slight swelling in these cases round the patella; but when this is present, inquiry should be carefully made respecting the previous treatment, as blisters will readily induce this appearance of effusion. Crural neuralgia is often a symptom of diseased hip, the pain being referred to the knee. We remember a case of this kind in which for two years the medical treatment had been directed exclusively to the knee, with the effect of inducing the suspicious tumefaction we have mentioned, but the result of the case showed that the knee was in no degree implicated, but the hip only.

*Acrodynia:* Neuralgia of the upper extremities. Professor Romberg agrees with Andral in viewing this epidemic as a neuralgia of the palmar and plantar surfaces. It prevailed in Paris in 1828 and 1829. The symptoms scarcely admit of this view; for although the pricking sensations in the skin were followed by partial anesthesia, yet there was exfoliation of the cuticle, and a change in the coloriferous glands, showing an apparent approach rather to an exanthem implicating the tactile and chromatogenous apparatus than the proper neuralgiæ; unless indeed we call any disease by that term in which sensibility is increased or diminished.

*Cervico-brachial neuralgia.* This, which is a more important class, is left altogether unnoticed by our author. These affections correspond in all points to the neuralgia of the lower extremities. It is stated by Valleix that the ulnar nerve is that most usually affected. There is, however, an affection of the shoulder which we think of more importance if not so frequent. We allude to a neuralgia closely analogous to sciatic neuralgia. As the gluteal are wasted in the latter, so is the deltoid in the former; in the one, the hip-joint is fixed, in the other the shoulder-joint; in the sciatic neuralgia, the lumbar vertebræ are deflected, in the humeral, the cervico-dorsal. In inveterate examples of the latter, the disease extends itself to other nerves, as of the arm and fore-arm. The intercostal muscles, the serrati and the latissimi dorsi, are also gradually implicated, and at last waste and are paralysed. This neuralgia is manifestly dependent upon a structural change in the central axis itself, gradually extending from a small diseased point until it implicates the nerves not only of the upper extremity, but also of the neck, head, and face.

We must pass over the remaining neuralgiæ of the sensitive nerves, namely, *mastodynia*, *pruritus*, *formicatio*, *ardor*, and *algor*. They are interesting rather physiologically than pathologically. Neither do the muscular neuralgiæ, namely, *anxietas tibiarum* and local spasms, present anything worthy notice.

*Vertigo*. There is much novelty in the notion which classes vertigo amongst the hyperesthesiæ of the muscular system. The essential characteristic of vertigo, according to Professor Romberg, is the sensation of *apparent* motion (as opposed to *real*,) or of apparent postures. This sensation, he argues, can be derived only from the sensitive nerves distributed to muscles. In proof he mentions the perception of the degree of resistance which the sole of the foot experiences in walking, of the weight of bodies when balanced in the hand, and of the direction in which the limbs are moved. The blind, for example, know when the arm is extended horizontally, or lifted up, as well as those who can see. Prof. Romberg's views are certainly ingenious, but we think he confounds *ideas* with sensations. The estimation of a weight by the hand, for example, is a complex mental act, differing *toto cœlo* from the pain of spasm; it not only presupposes a knowledge of weights, but it assumes the power of comparison. And after all, the illustration is invalid, for it is at least as reasonable and more philosophical to suppose that the sensation of resistance and of weight is obtained through the nerves of touch, nerves of special sense. On this supposition the amount of compression exercised on the nerves between the bones of the hand and the body indicates the weight. In all cases of complete cutaneous anesthesia, the power of estimating weight is lost, because this compression is not felt. It may certainly be objected that the muscular and sensitive nerves are paralysed with the cutaneous. But is there any case extant in which the muscular nerves were anesthetic and not the cutaneous at the same time? We believe not one. Another powerful argument against the views of our author, is derived from the fact acknowledged by himself, that vertigo (as defined) is followed by combined movements, which are independent of the will. Viewed in this way, they are analogous to the reflex movements excited on tickling the soles, the axillæ, &c. When the impression on the sensorial centres is not sufficient to excite movement, it excites the sensation of simple vertigo only; but, when more intense, actual movements follow. This we conceive happens in the curious class of diseases, in which there is rotation in the long axis of the body, or an irresistible propensity to run forwards or backwards, or to climb, &c., as occurs in epilepsy, hysteria, catalepsy. The sensation of vertigo only may be excited by looking from a height, or it may be so intense as to excite reflex *action*, and the individual is irresistibly impelled to precipitate himself forwards, and so not only risk his neck but a verdict of felo de se, and the forfeiture of his insurance and policy. The whole of the movements alluded to, and we may add the *imitated* movements, are in reality dependent upon another principle as yet but partially developed. We allude to the doctrine that there is a *peripheral sensorial development within the cranium and vertebral column as well as without; and that the action of ideas* [?] *developed on this internal periphery may be reflected upon the motor part of the ganglia and nerves, and motion follow, just as when impressions derived from the external periphery and transmitted to the motor part of the central ganglia are followed by common reflex movements, whether simple or associated.*

*Causes of Vertigo.* Professor Romberg's arrangement of the causes of vertigo is to us exceedingly unsatisfactory. They may, we think, be arranged under four heads. In the first are those which induce a change in the capillary circulation of the brain; as long-continued recumbency, bloodletting, disease of the heart, compression of the carotids, obstructed circulation through the lungs, sleep during febrile affections, cerebral irritation and congestion. 2. Changes in the composition of the blood, induced either by poisons taken into the circulation, as alcohol, camphor, belladonna, &c.; or by the retention of the excretions consequent on visceral disease. 3. Changes caused by cerebral sensations, as looking from a height, looking at a body in rapid motion, especially if rotatory, or even at a body in motion parallel with the plane of the horizon, &c. Purkinje's researches illustrate this class. 4. Structural changes in the encephalon, particularly in the cerebellum and its commissures; a class illustrated by the vivisections of Flourens, Majendie, Bouillaud, Kraus, and Hertwig, and the pathological researches of Greding, Serres, Toulmouche, Andral, and others. The causes of vertigo being so numerous and so widely different, it is mere pedantry in our author to devote one whole page to the treatment.

*Hyperesthesiæ of the pneumogastric.* The symptoms of these vary as the organs to which the branches of the nerve are distributed. There are, however, some general symptoms in which they resemble the cutaneous hyperesthesiæ. There may be: *a*, *Algor.* Professor Romberg has had hysterical patients who complained of a feeling of coldness in the bronchial tubes, although the air inspired was warm. *b*, *Ardor*, as in pyrosis. *c*, *Pruritus.* This is experienced in the bronchial tubes in bronchitis; and, according to our author, occurs as an uncomplicated affection. Pruritus of the external meatus auditorius from hyperesthesia of the auricular branch of the pneumogastric is sometimes observed, and is accompanied by cough and vomiting. This connexion between the ear and the stomach and lungs is not sufficiently remembered by modern practitioners. Arnold mentions an interesting example of chronic vomiting in a child, which long resisted all curative means, but which was effectually removed by removing a bean from each of the child's ears, that had slipped in while at play. Cassius Medicus has for one of his problems: Why does irritating the ears, as for example, with a speculum, cause sometimes a cough, just as if the trachea was irritated?

*d*, Neuralgia of the gastric branches appears as gastrodynia, and like tic douloureux is accompanied by altered secretion (pyrosis.) Not long ago, Dr. Graves published a case of neuralgia of the larynx.*

Hyperesthesia of these organs reacting upon the central ganglia will also induce a morbid exaggeration of certain instinctive feelings; when the pulmonary branches are affected, there is a feeling of the need to respire as in dyspnœa; when the gastric branches are implicated, bulimia and polydipsia are excited.

There is a definite hyperesthesia of the vagus, to which Prof. Romberg makes no allusion, namely, the pain and vomiting excited in some nervous patients, especially the hysterical, by the *smallest portion* of *animal* food. It has been termed *kreatic nausea,* and is a sufficiently well-marked disease to merit special investigation. These affections are often arthritic. We have a gouty patient who is always warned of an approaching fit by an insatiable thirst, and which continues until some joint is affected.

The sensual hyperesthesiæ. Professor Romberg classes spectral illusions among the hyperesthesiæ opticæ. In doing this, he utterly abandons his own principles of arrangement. Neither increased nor diminished sensibility of the optic nerve can be considered as in any way connected with spectral illusions; their site is undoubtedly cerebral. On the other hand, he makes no allusion to that morbid sensibility to certain colours (*chromatophobia*) observed occasionally, and corresponding to the congenital insensibility to particular colours long known to pathologists as an hereditary defect in the individuals termed *idiopts* by Mr. Whewell. There is little else worthy notice under this head.

Hyperesthesiæ of the sympathetic nerves. Professor Romberg allocates the diseases under this head in the plexus of the sympathetic, giving no reason whatever for that arrangement, or any dissections in support of it. We will, however, notice them as we find them.

*Hyp. plexus cardiaci. Angina pectoris.* Professor Romberg contravenes the old, and, we may add, now exploded opinion, that this disease originates in structural disease of the heart or coronary arteries. It is manifestly a pure neuralgia, of centric origin, but its seat is by no means in the cardiac plexus exclusively. The sensory portion of the cervical cord, or of the respiratory ganglia must more properly be considered the true seat of this painful disease. If a case be watched *ab initio* it will be found that at first the heart is unaffected. The spasm is confined to the respiratory muscles, and the pain is dependent on that spasm, and seated in the muscles, principally the triangularis sterni and diaphragm, although, in one case which came under our notice, the pectorales were also the seat of severe pain—a scraping, tearing sensation—and were felt by the patient to be hard and tense during the paroxysm. As the disease advances the pneumogastric and nerves of the cardiac plexus become implicated, and there is ventricular excretion of gas, weak fluttering pulse, and more urgent dyspnea. But the cutaneous nerves of the skin have also become proportionally affectible, and a breath of cold air, or even pressure on the sternum, will induce a paroxysm.

Arthritis, hysteria, and spinal neuralgia are named by our author as

* Dublin Journal of Med. and Chem. Science, vol. xiv, p. 372.

the most frequent causes of the disease. We believe that hysteria, taken in its most extended sense, is rarely a cause. Women are peculiarly exempt from this and certain diseases of the thoracic viscera. Spasmodic asthma is almost the exclusive plague of men : ten elevenths of the examples of angina pectoris are to be found amongst males,* and cardiac and arterial aneurism in about the same proportion. The arthritic ossification of the coronary and large vessels of the heart is almost peculiar to men, and formerly supposed to be the proximate cause of angina pectoris, probably only a predisposing cause. Indeed it may even be fairly argued that, like other structural changes of the heart, it is a sequel of the affection.

*Treatment of angina pectoris.* The ossification of the heart and large vessels just alluded to, plainly points to arthritis as one of the casual indications. In these cases Professor Romberg recommends issues and setons in the region of the *heart*—a recommendation quite sufficient to establish the opinion we have already expressed, that Professor Romberg has no idea of the mode in which the arthritic diathesis is connected with the origin of arthritic neuroses. Having already shown that it is in accordance with analogy to suppose that arthritis, whether acute or chronic, may attack the serous membranes of the nervous centres, and through these induce any form of neurosis, the locality of the issue or seton should be the cervical region, and not the cardiac, as recommended by Professor Romberg. In other respects the curative treatment is not different from that generally adopted in England. As a palliative during the paroxysm, Professor Romberg has found that the *inhalation* of sulphuric or acetic ether affords the speediest relief.

*Hyp. plexus solaris. Cœliac neuralgia.* This affection, as described by our author, is the same as that termed cardialgia, or, popularly, "the spasms." It sometimes assumes an intermitting type. The corresponding spinal nerves and the branches of the pneumogastric are manifestly implicated. Professor Romberg presents us, in his own person, with an interesting illustration of the remarks we have just made regarding the arthritic neuroses. He says, "Arthritis predisposes: I have endured this affection myself, as a precursor of my first attack of gout, and retain a lively recollection of its annihilating sensations and pain, as if the epigastrium was grasped by claws." It is probable that sometimes carcinomatous degeneration of the stomach is only a sequel of this affection.

*Hyp. plexus mesentericæ. Lead colic.* There is nothing of moment stated by our author regarding this neuralgia, excepting two facts, which necessarily derange his nosological arrangement. The one is, that the spinal cord plays an important part in the affection ; the other, that pathological anatomists have failed to detect structural change in the ganglia or branches of the sympathetic

Hyperesthesiæ of the hypogastric and spermatic plexus comprise, according to our author, dysmenorrhea and irritable uterus and testis. His pathology, we need not say, is altogether hypothetical.

Order II. Hyperesthesiæ of the Central Organs. By central organs are here meant only the brain and spinal cord. Professor

* Cyclop. Pract. Medicine, art. Angina pectoris.

Romberg discusses the recent experiments of Majendie, since corroborated by Volkmann, with reference to the sensibility exhibited during vivisections, by the anterior columns and the roots of the motor nerves. It now appears to be established that this is due to the *nervi nervorum*, or the twigs derived from the posterior or sensitive roots, and distributed within the substance of the nerves and to the anterior columns. It is to these nerves that our author refers the pain in myelitis, spinal meningitis, cancerous or neuromatous degeneration of the cord, &c. Examples of these are adduced. It is worthy notice, however, that pain in the spinal cord is not even mentioned in the history of some of these cases, and in others there is no reason given why the pain in the back should be referred to the spinal cord rather than to the spinal muscles and ligaments. And when we come to our author's views respecting spinal neuralgia, as he designates the spinal irritation of English writers, we find that he leaves the subject altogether, and confounds cutaneous neuralgia dependent upon centric disorder with what we should suppose he intended to treat of, namely, neuralgia of the spinal cord itself. He proceeds to detail how, when the cervical region is affected, the pain extends over the skin supplied by the second and third cervical nerves; how, when the upper dorsal portion is the seat, there is *pleurodynia neuralgica*; how, when the lumbar portion is affected, there is tenderness of the abdomen and along the spinal column, pain in the lower extremities, and colic from time to time. Nor are the viscera and consequently the vagus and sympathetic nerves in these various regions exempt. Spasm of the pharynx, larynx, and œsophagus, singular coughs, hiccough, syncope, palpitation, ventricular and intestinal excretion of gas, constipation, ischuria, diabetes, &c., accompany the various forms of the so-called spinal irritation. Now, these phenomena admit of easy solution when properly considered. The cause of the hyperesthesia is centric, and the symptoms are in direct proportion to the extent and degree of this centric affection. If the sensory spinal nerves only are affected, we shall have the true neuralgia described by M. Valleix.* The cause of the hyperesthesia being centrical, all the sensitive twigs passing through the affected portion of the cord will be readily affected by stimuli, and those which ordinarily give no pain, but sensation only, as the tactile twigs, will now be painful. We should therefore, *à priori*, expect that the neuralgia here described will be found to attack nerves exposed by their anatomical distribution to mechanical irritation, or which normally are more sensitive than others. The nerves that traverse bony canals or foramina, or that wind round bones, or have to penetrate muscles, are of this class: so also the special points of sensibility described by Weber, as the middle of the sternum, or of the spine. The trophical nerves are also affected in spinal neuralgia. Tartar-emetic ointment rubbed along the spine in a case of this kind will bring out pustules at the tender points, long before they appear on the unaffected surfaces.

*The treatment* recommended is that usually adopted by English physicians. The principle of endermic medication is more useful in these cases than in others. We have found useful a liniment composed of belladonna extract ʒij, arsenical solution ℥ss, camphorated oil ℥iss, ap-

* British and Foreign Medical Review, vol. XIII, p. 138.

plied to the spine two or three times a day, and conjoined with salt-water douches; taking care, of course, that the *general* treatment suitable to the case be followed up. The irritation of the spinal nerves or ganglia being thus alleviated, the visceral functions are performed more naturally, and an opportunity is given to the system to right itself.

*Hyperesthesia of the brain. Dolor cerebralis.* "There is none," says our author, "among the diseases of the brain, in which there is not pain, with the exception of atrophy of the brain." (p. 161.) In the passage preceding this he observes: "It is of importance to remark that when a portion of brain is protruded through a fracture or opening of the skull, it is altogetber insensible to wounding or pressure, whether it be inflamed or projects from the skull as an indurated fungus." And yet he seriously discusses hyperesthesia of the brain proper, as though there were no membranes around it, highly sensible when inflamed, irritated, or compressed; and founds on this simple erroneous symptom of pain in the brain a consideration of the structural diseases to which it is liable. The pathology of cerebral tubercles, hydatids, tumours generally, cancer, abscesses, softening, induration, hemorrhages, meningitis, are discussed *seriatim*, and, to crown the absurdity, the question—Does the seat of cerebral pain indicate the locality of the disease?—is answered in the negative.

*Neuralgia cerebralis* or *hemicrania* is really a neuralgy of a spinal nerve or nerves, and out of place here. Professor Romberg has observed it to be hereditary. He cautions against the abuse of remedies in the treatment; recommends the recumbent posture, and avoidance of all stimuli during the paroxysm, and attention to the digestive organs in the intervals.

*Hyperesthesia psychica. Hypochondria.* This disease, in all its phases, is described in a happy and somewhat humorous manner. Our author defines it to consist in the excitement and continued maintenance of abnormal sensations by fixing the mind on the feelings. "The hypochondrist is the virtuosa of the sensitive nerves." To understand the secondary pathology of hypochondria it is necessary to refer to the laws of sensation we have before alluded to. These we need not discuss here; the practical results of these laws are of more importance. At a meeting of the Westminster Medical Society, held Dec. 21, 1833, Mr. Quain detailed one of these. A gentleman who had constantly witnessed the sufferings of a friend afflicted with stricture of the œsophagus, had so great an impression made on his mind (or rather brain) that after some time he experienced difficulty of deglutition, and died of spasmodic dysphagia. Our author justly observes that it is not only wearisome but dangerous to live with a hypochondriac. Professor Romberg, however, like all other writers, fails to give what we think the true or primary pathology of hypochondria. To term it a psychical hyperesthesia is a very compendious but very unsatisfactory mode of explaining it. In pathological language, psychical means cerebral. That hypochondria depends on a local neurosis of the cerebrum is manifest from several considerations. Our own observations corroborate the opinions of Stahl, Whytt, Tode, and Weikard as to the connexion between hypochondria and gout. We have seen it as an acute disease ushering in an attack of gout, and also as a symptom of abdominal congestion in gouty subjects. The morbid anxiety which characterizes hypochondria is in fact but a modification of that morbid state usually termed mental depression, and which has various causes and various phases between the melancholy of the bilious dyspeptic, and the despair of the suicide. We see this morbid anxiety in the different forms of insanity: in one, concentrated on a provision for offspring; in another, on a provision for self. We see it also in a sequel of hemiplegia and of delirium tremens, and of depressing agencies, whether mental or corporeal. It accompanies paroxysms of hysteria and it is often witnessed in the mesmerized. All these facts point to the conclusion that the leading symptom of hypochondria is dependent upon a local cerebral neurosis. This conclusion must necessarily modify the treatment, and in fact we have been most successful in treating hypochondria as a cerebral affection, and according to its causal indications. In *all* cases, the shower-bath will be found of service, and in those connected with a gouty diathesis, small doses of iron and colchicum (or aconite) in combination with dietetic means. When a sequel of mental agencies of a sorrowful or harassing character, travel and an active life are absolutely necessary for cure.

THE ANESTHESIÆ. Professor Romberg introduces these by recurring to the general laws of sensation before mentioned. He lays down the synergies: 1, between the cutaneous and sensual nerves; 2, between the sensitive and motor; and 3, between sensitive and trophical nerves. In treating the cutaneous anesthesiæ, he distinguishes between the peripheral and central. In considering the anesthesiæ of the muscular nerves, he falls into the same mistake as when treating of vertigo, making his confusion on this point worse confounded. Some interesting remarks are made on anesthesia of the vagus, especially with reference to

*Respiratory anesthesia.* This affection is characterized by insensibility of the bronchia, and the absence of the urgent demand for air. Professor Romberg first noticed it in that form of Asiatic cholera accompanied by asphyxia. An example of the disease as it occurred in a child is given. In this case the respiration was sonorous and difficult without any of the usual expressions of anxiety in the countenance; there was no violent action of the respiratory muscles, and no cough. After death, the *glandulæ concatenatæ* in the neck were found to be filled with tubercles, and had suppurated, surrounding and compressing the vagus, so that at one point it appeared plainly flattened. If a whelp be placed in the receiver of an air-pump, it breathes quicker and quicker, gasps more and more, and at last dies asphyxiated. But if the two vagi be cut at the same time, the animal under the same circumstances gives no signs of distress, and dies quietly in thirty or forty minutes.

It is probable that indigestion or anorexia is occasionally dependent upon a paralysis of the gastric branches of the vagus.

THE SENSUAL ANESTHESIÆ. These are next discussed, and to make his arrangement complete, Professor Romberg discusses spinal and cerebral anesthesiæ, in the same loose manner as the hyperesthesiæ of the central axis.

THE MOTOR NEUROSES. Professor Romberg divides the motor neuroses into two great divisions: the *hypercineses* or spasmodic, and *acineses*,* or

* Hypercinesis is compounded of ὑπὲρ and κίνησις, from the verb κινέω, I move. Acinesis from *α* privative and κίνησις.

paralytic. He also fully adopts the anatomical and physiological views of Dr. M. Hall and Prof. Müller. These we need not repeat.

THE HYPERCINESES are divided into two orders :

ORDER I. Spasm from excitement of the motor nerves as conductors.

GENUS 1. The cerebro-spinal nerves in their peripheral or central course. Convulsive tic, trismus, strabismus, nystagmus, torticollis, spasms of the fingers and of the muscles of the lower extremities.

GENUS 2. The sympathetic nerves, of which it is not yet known whether their ganglia possess the central quality of irradiation. Spasms implicating the respiratory muscles, the digestive apparatus, the intestinal canal and genito-urinary systems. [These, we suppose, belong to this genus, for our author gives us no means of learning his opinion.]

ORDER II. Spasms from excitement of the central apparatus.

GENUS 1. Of the spinal cord.

*a.* Spasms depending on the cord as the combining apparatus, raphania, chorea S. viti.

*b.* Spasms from exalted reflex action. Hysteria, tetanus, hydrophobia.

*c.* Spasms from abnormal evolution of the motorific agent. Tremores, namely, T. *potatorum*, T. *senilis*, T. *febrilis*. Paralysis agitans.

GENUS 2. Of the brain.

*a.* Antagonistic spasms or vertiginous movements, i, longitudinally ; ii, anteriorly; iii, posteriorly ; iv, alternately antagonistic.

*b.* Coordinated spasm.

*c.* Psychical spasms. Eclampsia, E. *toxica*, E. *parturientum*. Epilepsia.

As our allotted space is nearly exhausted, we can only glance at Professor Romberg's views respecting these hypercineses, especially those of the first order, and it is the less necessary that we should do more, because the diseases placed in that order are for the most part connected with the hyperesthesiæ or anesthesiæ already discussed, and as regards the general pathology and treatment, do not generically differ from the latter. The laws of isolated conduction, of central irradiation, and of synergy with the other classes of nerves, apply to the motor equally as to the sensitive ; while the anatomical distribution of the two is different only in special instances. We shall notice the more prominent and practical points only.

*Contracted hip.* We have already given the diagnosis of sciatica simulating hip-disease, and we would caution the practitioner against mistaking spasmodic contraction of the psoas, iliacus and quadratus lumborum muscles for morbus coxæ In the contracted hip, the limb is shortened and attempts at extension excite pain in the nerve. The tendons of the pelvic muscles, and the muscles situate externally may be felt like tense cords.

*Spasmodic asthma*, or spasm of the bronchi. To palliate the attacks, Professor Romberg recommends that the gastric branches of the vagus should be acted on. He has found small doses of ipecacuan, and the application of cold very useful. Ices give immediate relief; cold-water clysters are also beneficial.

*Spasm of the respiratory muscles.* Hiccup, yawning, sneezing, laughing, strange spasmodic coughs, &c., are under this head. For prolonged hiccup, cold affusion is advised. Cruveilhier cured two obstinate cases by bending the head back, and pouring the water into the mouth and nostrils of the patients. Spasmodic sneezing may be relieved by emetics.

*Spasm of the sphincter ani.* Professor Romberg mentions constipation as a symptom of this spasm. We have observed both constipation and diarrhea in cases of this kind. We quite agree with him that division of the sphincter is the surest and quickest mode of cure.

Passing on to the spinal affections, we find that the first treated of is that induced by using spurred rye as food. (Raphania.) This affection resembles beriberi in many points, and it is rather surprising that Prof. Romberg has not observed the analogy. The disease is scarcely known in England.

*Hysteria.* Professor Romberg observes that next to exalted reflex excitability, the psychical state indicated by the powerlessness of the will is the most remarkable in hysteria. He adopts Sir B. Brodie's views in this respect, observing that the hysterical motto, "I cannot help it," is often heard. In discussing the causes of hysteria, he alludes to, and adopts the views of Dr. Laycock, as to the ovarian origin of the affection, and as to the extensive influence of the ovaria on the nervous system.

The question of neurological sympathies is illustrated in a limited degree only by the recent neuro-physiological discoveries. We can easily imagine how the testes and ovaria may direct an incident action on the lumbar spinal cord, and excite a reflex action; but how can we explain their influence (as demonstrated by Dr. Laycock,) upon the whole respiratory system, and upon portions of the brain? as, for example, when certain mental powers are excited into operation by ovarian influence? We should have been glad to have seen this matter discussed by Professor Romberg.

He quaintly refers to the morbid *cunning* developed in patients by this ovarian influence. "*Mulieri ne mortuæ quidem credendum est*" is, he says, particularly applicable to the hysterical.

In discussing the general treatment, Professor Romberg lays much stress on the utility of reading aloud in promoting the cure, and we think with some reason. He states also that he knows of no palliative more effectual against the yawning and other respiratory spasms than this. He advises that whatever medicines are prescribed, they should be pleasing in form and small in quantity. "Dirty-looking eight-ounce mixtures," he observes, "and boxes crammed full of pills may please the hypochondriac, but make the hysterical obstinate and anxious, and create ennui."

*Tetanus.* This disease has been carefully considered by our author, and the best writers consulted. The true characteristic of the disease is reflex irritability in its most intense form. Similar motor disturbance and like spasmodic affections are observed in meningitis, but that despotic power (to quote Professor Romberg's phrase) is wanting which makes the entire muscular system of an athlete dependent upon some slight irritation of a small point on the skin; and this also constitutes the difference between tetanus and spasm of groups of muscles, as the masticatory, even when dependent upon like causes. On these grounds our author argues that the usual division of tetanus into trismus, orthotonus, emprosthotonus, &c. are trivial; and also that the distinction of inflammatory and spasmodic, idiopathic and symptomatic are inadmissible. The nosological division of Mr. Curling into acute, acute inflammatory, and chronic, is also quite erroneous. *T. rheumaticus* and *T. toxicus* are the two forms which are dependent upon a primary affection of the spinal cord, there being no irritation of the peripheral nerves. Rheumatic tetanus is rather a disease of warm climates than of cold, and is scarcely less incurable and fatal than the traumatic form. No reason is given for applying the term rheumatic to what is usually termed idiopathic tetanus; indeed the notice of the disease altogether is very meager and unsatisfactory.

*Hydrophobia.* Strychnine acts directly on the spinal cord; the poison of rabies is limited to the axis of the respiratory system—the medulla oblongata. The irritability of the skin observed in traumatic tetanus is here concentrated on the mucous and cutaneous surfaces, supplied by the pneumo-gastric and fifth pair, and is often so intense, that the contact of the air in common respiration induces bronchial or pharyngeal spasm. In prolonged cases, the spinal motor nerves are affected, and generally tetanus supervenes; but usually the impeded nutrition and respiration are quickly followed by prostration and asphyxia or apoplexy.

Hydrophobia is one of the most undoubted of the *opprobria medicorum.* The seat of the disease itself sufficiently explains why, and we think Professor Romberg has done well to turn his attention to the modes of prevention rather than of cure. It does not appear that the susceptibility to the action of the poison is great. Hertwig states that of 59 inoculated dogs, only 14 were affected with rabies, or 23 per cent. In 60 authentic cases, the shortest period of incubation was 14 days, the longest 9 months, the medium 4 to 7 weeks. In 17 authentic cases we have transcribed, the shortest period was 3 weeks, the longest 18 weeks, the average 58 days. Trolliet states that of thirteen persons bitten on the same day, by a rabid wolf, hydrophobia appeared in six in from 15 to 30 days, in four in from 30 to 40; in two the period was from 40 to 53, and one exhibited no symptoms for three months and eighteen days. In dogs, the disease, according to Hertwig, appears within 50 days after the bite. Depressing circumstances, especially mental affections, favour the outbreak of the disease. Although the cure of hydrophobia be utterly hopeless, and no antidote has yet been discovered, the prophylaxis is full of hope. Excision of the bitten part is the surest mode of prevention, and scarcely inferior is the destruction of it by caustics; Mr. Youatt advises that the nitrate of silver be used for this purpose; Rust and others recommend the caustic potash. Ligatures and cupping-glasses may be made auxiliaries to these, but should not be trusted alone.

*Psychical spasms.* We pass the class of motus vertiginosi to notice these, the "imitated movements" of English writers, and on which as well as on the vertiginous affections, animal magnetism may be expected to throw some light. The subject is one of the most important in cerebral physiology, and has a decided bearing on education, morals, and social economy. Epidemic fanaticism, tarantism, the leaping ague of Scotland, and other well-known examples of this kind of disease are mentioned. This imitative propensity appears sometimes as a chronic affection. We have been consulted respecting a girl of five years, who when spoken to, gave no answer, but repeated what was said to her like an echo. Professor Romberg who terms this form "echo," states that he has observed it in many individuals, and in variously diseased states of the brain. A case has been published, in which an adult had irresistibly imitated from early infancy all the muscular movements of individuals about him. If this dotterel-like propensity was forcibly restrained, he complained that his heart and brain were vexed.

*Eclampsia parturientum.* In this affection Professor Romberg joins with the English and American writers in recommending copious bleeding, opium, and immediate delivery. If the convulsions resist this treatment, counter-irritants to the nuchæ, local depletion, the cold shower-bath, turpentine and assafetida clysters, &c. Dr. Denman's plan of dashing cold water in the face is we think perfectly in accordance with the pathology of the disease. The convulsions are reflex ; they come on while the head of the child is pressing on the os uteri, and commence with the laryngeal muscles. Respiration is suspended, cerebral congestion follows on the cardiac and pulmonary congestion, and then general convulsions supervene.

Puerperal convulsions are comparatively rare. Madame Lachapelle states that they occurred to 68 only of 34,000 parturient females; Mayer had only five cases in 2500; but Merriman had more, 48 in 2000.

*Epilepsy.* Professor Romberg has furnished a good description and history of epilepsy in all its forms and complications. An interesting case is detailed in which various of these complications occurred in a regular sequence. We think it will interest our readers, especially the physiological and mesmeric, but regret that our notice is already too lengthened to give it in detail. The subject of it was a girl, aged twenty-eight years, has for twelve years been subject to epileptic attacks, (the consequence of a fright,) during which there is a maniacal condition. In the intervals, the lower extremities are partially paralysed, the patient being unable to rise from her seat, or to go scarcely a few steps, even when supported by two persons. She has also for two years been affected with an obstinate quartan. The details, the correctness of which is we think indisputable, affords matter for curious speculation. One inference may be fairly drawn from them, namely, that our knowledge of the functions of the hemispherical ganglia is almost *nil.*

One hundred and fifty quarto pages of Hennen's 'Analecta literaria Epilepsiam Spectantia,' published in 1798, are filled with the remedies for epilepsy. The experience of forty-five years has added to their number ; but probably there are not two of the whole that can be prescribed with any confidence; we shall not therefore review them. Professor Romberg cautions against moderating the convulsions during the attack, or limiting their duration ; the more complete the paroxysm, particularly after a long interval, the greater is the relief of the patient. There is some truth in this. Another caution is necessary. A ligature to a limb, especially if there be an epileptic aura from it, will prevent a paroxysm ; but if the use of the ligature should be forgotten or neglected after having been used awhile, there is a great danger of a fatal accession. Dr. Townsend related a case of this kind to Dr. Graves. The fits were extremely frequent, (five in a night,) and were warded off for four months, by the means of a stick and a cord round the leg. After that period, the patient left off the use of the latter, and died in the first paroxysm.

We have now finished our review of this the first volume of Professor Romberg's work. It is imperfect in several points, as we have shown, but it is not without merit. We feel curious to see the next volume, and learn our author's views on the psychical neuroses. If he be inoculated with the usual metaphysics of the German school, they will not possess much value. Should, however, future neuro-physiological researches be as successful as the past, and these be worked up by our author, his first volume will need rewriting by the time his second is finished.

